# Impact and cultural acceptance of the Narrative Exposure Therapy in the aftermath of a natural disaster in Burundi

**DOI:** 10.1186/s12888-018-1799-3

**Published:** 2018-07-18

**Authors:** Anselm Crombach, Sebastian Siehl

**Affiliations:** 1 Department of Psychology, University of Konstanz, Konstanz, Germany; 2 Department of Psychology, University Lumière of Bujumbura, Bujumbura, Burundi; 3 Non-Governmental Organization Psychologues sans Frontières Burundi, Bujumbura, Burundi; 4 Non-Governmental Organization vivo international e.V., Konstanz, Germany; 5 Department of Cognitive and Clinical Neuroscience, Central Institute of Mental Health, Medical Faculty Mannheim, University of Heidelberg, Mannheim, Germany; 6 Graduate School of Economic and Social Sciences, University of Mannheim, Mannheim, Germany

**Keywords:** Narrative Exposure Therapy, Natural disaster, post-conflict, Trauma-focused interventions, Psychological assistance, Burundi

## Abstract

**Background:**

In the aftermath of natural disasters, affected populations are at risk of suffering from trauma-related mental health disorders such as posttraumatic stress disorder (PTSD) or depression. Particularly in poor post-conflict regions, these mental disorders have the potential to impair the ability of individuals to move on with their lives. We aimed to evaluate the feasibility, cultural acceptance, and effect of a trauma-focused psychotherapy, Narrative Exposure Therapy (NET), in the aftermath of a flood disaster in Burundi.

**Methods:**

Fifty-one individuals who were living in emergency camps overseen by the Burundian Red Cross in the aftermath of a flood disaster, and who had lost homes and close relatives, were invited to participate in semi-structured diagnostic interviews. Trained Burundian psychology students conducted these interviews, and six sessions of NET were offered to the 15 individuals most affected by trauma-related symptoms. An additional group of psychology students, blind to the treatment conditions, conducted three and 9 months follow-ups with them including also 25 participants who had reported significant but less severe trauma-related symptoms, assessing mental health symptoms, acceptance of NET, stigmatization due to trauma symptoms, and participants’ economic well-being.

**Results:**

Between baseline and 9-months post-intervention assessment, symptoms of PTSD (*Hedges’ g* = 3.44) and depression (*Hedges’ g* = 1.88) improved significantly within participants who received NET and within those who received no treatment (*Hedges’ g*_*PTSD*_ = 2.55; *Hedges’ g*_*depression*_ = 0.72). Furthermore, those who received NET felt less stigmatized by their participation in the intervention than by the trauma-related mental health symptoms they experienced. Overall, participants reported that they would be willing to forego as much as 1 month’s worth of income in exchange for receiving trauma-focused interventions in the months following the disaster.

**Conclusions:**

Individuals severely affected by trauma-related mental health symptoms might benefit significantly from NET in the aftermath of natural disasters, while less affected individuals seem to recover spontaneously. Despite significant challenges conducting NET in emergency camps in the aftermath of natural disaster in a post-conflict country, such interventions are feasible, appreciated and might have long-lasting impacts on the lives of survivors if conducted with due respect to participants’ privacy.

**Trial registration:**

UKCR2014, the 19.06.2014, retrospectively registered.

## Background

In the aftermath of natural and human induced disasters, affected individuals struggle to come to terms with the often-horrifying consequences. Particularly in low-resource crisis and post-conflict regions, the path to recovery is often threatened by economic loss, disrupted community or family systems, and health impairments. Gulliver, Zimering, Carpenter, Giardina, & Farrar [1] describe a disaster “as a natural or man-made event, that negatively affects life, property, livelihood …” (p. 25) and which leads to an increased incidence and relapse of mental health disorders. Furthermore, the life-threatening nature of the disaster, the loss of loved ones, and, in some cases, the irreversible physical impairment of affected individuals further contributes to the risk of developing mental health disorders. Such disorders might prevent victims from benefiting sustainably from material aid that is often provided as part of organized relief efforts. In their extensive review, Galea, Nandi, & Vlahov [2] describe posttraumatic stress disorder (PTSD) as the most frequently occurring psychological disorder appearing after the experience of a natural disaster, with prevalence rates ranging from 5 to 60%, with higher rates in areas more severely affected by the disaster. According to Galea et al. [2] the strongest predictor for developing symptoms of PTSD is the extent of exposure to the disaster, with higher risks for individuals with high exposure. In addition, research on the *building block* effect, i.e., a dose-response relationship between the number of experienced traumatic event types and PTSD symptoms, emphasizes that prior traumatic experiences have a severe impact on mental health and substantially increase the risk of suffering from trauma-related mental disorders (e.g., [3, 4]). Indeed, in the aftermath of the 2004 tsunami, a study conducted in Sri Lanka identified previous traumatic exposure, severity of exposure to the natural disaster, and loss of family members as significant predictors of PTSD symptoms in children, and consequently found elevated prevalence rates in violence-affected crisis regions compared to more stable regions [5].

The devastating impact of mental health disorders has been increasingly recognized as a crucial risk factor potentially causing long-term human misery and impairment in health and economic productivity in low-income countries [6]. Even though mental health disorders have been identified as a major obstacle to successful recovery, the best way of responding to psychological needs in the aftermath of humanitarian disasters in crisis regions has been subject to highly controversial discussions in recent years. Experts disagreed strongly regarding the implementation of psychotherapeutic interventions to address PTSD. Some have argued that the concept of PTSD is culture-bound and irrelevant outside the context of Western cultures. They stressed that attaching diagnoses and offering treatment might be stigmatizing, and might not sufficiently reflect the emotional suffering and daily worries of individuals in such contexts [7]. Those experts argued that reducing daily stressors, such as financial hardship or barriers to resettlement [8], through psychosocial interventions would be the key to address mental health issues in these circumstances [9]. Furthermore, they emphasized the prominent role of daily stressors in predicting and maintaining mental distress [8, 10].

Others argued, however, that correlations between a low socio-economic status (SES) and increased vulnerability for mental disorders, such as symptoms of depression or PTSD, are at least partially explained by the impaired functionality of affected individuals and entire communities. For instance, a study with former child soldiers suggested that symptoms of PTSD are associated with a reduced openness to reconciliation and elevated feelings of revenge [11], thereby impairing trust and collaboration in the community. Furthermore, research indicates that the degree to which potential daily stressors are perceived as stressful depends on the mental health condition of an individual, as a bias towards a negative evaluation and interpretation of a situation is a core element of anxiety disorders and depression [12]. For instance, traumatized individuals also feel threatened more easily and hence might perceive daily struggles as more stressful and intimidating than individuals not suffering from trauma-related disorders. Furthermore, daily stressors are likely to trigger feelings of fear and helplessness, which are then exacerbated by feelings, cognitions, and interoceptive impressions related to previous traumatic experiences [13, 14]. Therefore, many clinicians emphasize the necessity of addressing trauma-related disorders with evidence-based methods that are evaluated in the specific context in which they are being utilized and can be applied by lay-counsellors instead of aiming to stabilize perpetually unstable environments [7, 15].

Narrative Exposure Therapy (NET) has been developed as a standardized, short-term treatment of PTSD for survivors of war, domestic violence, torture, and natural disaster [16]. NET embeds the principles of testimony therapy [17], prolonged exposure therapy [18], cognitive-behavioural and client-centred psychotherapy into recent findings of neuro-traumatology. Extensive research demonstrated the effectiveness of NET in a wide range of contexts and populations with symptoms of PTSD and depression. Randomized Controlled Trial studies (RCTs) revealed positive treatment effects for adults and children who suffer from mental illness after the experience of multiple traumatic events [19–22], including natural disasters [23, 24]. NET has also been proven effective in a short form with only 4 to 8 sessions, making it a valuable tool in insecure and volatile environments such as in the aftermath of a flood disaster or within post-conflict countries [21, 25]. Such efficacy in unstable situations is compounded by the successful dissemination of NET to local laypersons, which has been shown in several studies [25, 26]. A similar reduction of symptoms in patients with PTSD was found in studies focusing more generally on exposure therapies outside of NET, although those participants received therapy provided by a highly experienced therapist or a trained local health worker [27–29].

Several reviews and meta-analyses provide evidence of the efficacy of NET in a broad range of cultures such as the Middle East, Central- and North Africa as well as Europe [25, 30–32]. Symptoms of PTSD were significantly reduced in all of the studies reviewed with moderate to high effect sizes. In general, the symptom severity continued to decline with longer time periods between the completion of NET and the follow-up. This continuous symptom reduction might be explained by long-term reorganization of memories and neuroplastic changes in the brain following completion of therapy [13, 33]. However, it has never been attempted to assess quantitatively the degree to which populations in African post-conflict countries value a trauma-focused intervention such as NET as an improvement of their lives. Assessing the value which affected populations attribute to these interventions seems particularly important in the aftermath of acute emergencies, including natural disasters. Such valuations may serve as a guide to institutions financing emergency responses in how to invest their resources regarding material and psycho-social assistance. An approach assessing acceptance is *Willingness to Pay* (WTP). WTP is defined as the maximum amount of money that an individual is willing to sacrifice in order to get a certain product or service [34]. Although, WTP was originally used for cost-effectiveness analyses, it can be successfully applied in evaluating treatments of PTSD [35] and depression [36]. Unützer et al. [37] concluded that WTP can be used to assess the value of treatments and reported in a sample of 615 American depressed primary care patients an average WTP of 9% of the participants’ monthly household income.

Another obstacle for psychological assistance that has to be taken into account in the aftermath of disasters is stigmatization. Goffman ([38]; pp.4–5) defines social stigma as “the phenomenon whereby an individual with an attribute is deeply discredited by his/ her society and is rejected as a result of the attribute”. Read, Haslam, Sayce, & Davies [39] indicated in their review that stigma might be related to biogenetic causal theories. The word “*illness*” for example triggers the perception of danger and unpredictability. A natural reaction that follows is fear and the desire for social distance. The result is that clients often face social rejection, dislike, or devaluation by others. Moreover, the belief that symptoms are self-inflicted [40] is widespread. Social stigmatization and isolation seems to be a phenomenon arising independent of cultural background [41] and is also found in individuals with symptoms of PTSD [42]. Hence social stigma can also occur by seeking treatment. Often people then decide not to seek adequate treatment, refrain from fully participating, or drop out [43]. Furthermore, research indicates that social disapproval impairs the recovery from trauma-related symptoms [44–46].

Burundi is a small country in Eastern Africa that has served as a battlefield for two ethnically-driven civil wars during the past 50 years. These civil wars, coupled with other political challenges, have served to decimate the country’s ability to cope with the effects of their frequent natural disasters. In February 2014, heavy rainfall flooded several districts of the capital of Burundi, Bujumbura. The resulting floods destroyed the livelihood of over 12,500 people, and killed at least 64 people, most of whom were children under the age of 10 [47]. In the wake of these events, we decided to conduct a feasibility trial regarding the implementation of NET as a trauma-focused intervention in the aftermath of a natural disaster in a post-conflict setting. We assumed that individuals suffering severely from PTSD symptoms in particular might benefit from such an intervention to prevent chronicity of symptoms, while less affected individuals might recover spontaneously once their living conditions became more stable. In addition to the efficacy of NET regarding mental health improvements, we also aimed to assess the fictional monetary value the survivors would attribute to receiving this intervention compared to their SES, and if they would feel more stigmatized by the treatment than by their mental health symptoms.

## Methods

### Participants

**Fig. 1:**
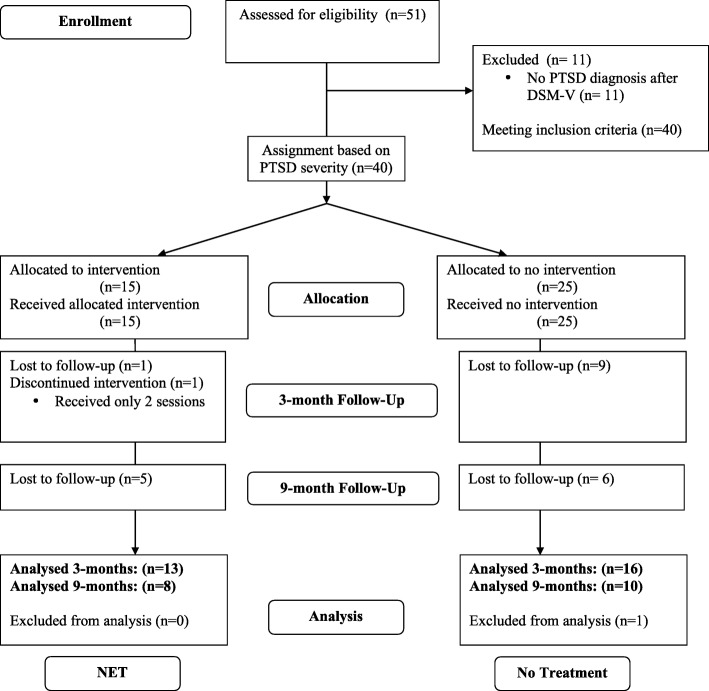
Consort flow chart

Initially, the project started as humanitarian aid project, assisting the Burundian Red Cross in three emergency camps established in response to the flood disaster in February 2014 in Bujumbura. The emergency camps were located in the districts Kinama, Kamenge and Buterere. Burundian Red Cross volunteers working in these camps identified 51 individuals who they considered to be severely affected by the disaster for the mental health experts of our team. Apart from their personal impressions of which survivors might need psychological assistance, they were encouraged to include individuals who had lost a close relative during the disaster, or individuals who were known to be having trouble sleeping, waking up screaming at night or reporting nightmares, and individuals who were socially isolated and did not engage with others in the close camp environments. During the initial diagnostic interviews, we found that 40 individuals suffered significantly from trauma-related mental health symptoms, i.e., they fulfilled minimum diagnostic criteria for PTSD. Those were enrolled in the trial (Fig. 1). Exclusion criteria for receiving NET were pregnancy, ongoing substance abuse and obvious psychotic symptoms. None of these applied to the recruited participants. At baseline, 40 individuals were included, with 15 participants in the NET group and 25 individuals in the no treatment group. At 3-months follow-up, we relocated and assessed 29 participants with 13 participants in the NET group and 16 participants in the no treatment group. At 9-months follow-up, we relocated and assessed 18 participants, with 8 in the NET and 10 in the no treatment group. Participants who were lost or who dropped out were excluded from respective statistical analyses. While we focused mainly on adults we also included one minor, aged 14 years old.

Participants were informed verbally that their participation in the initial interview and potential intervention would be entirely voluntary and that they could withdraw from it at any time and for any reason without facing any negative consequences. They were informed about the objective of the assessment and the intervention, potential benefits and risks, confidentiality, and that no monetary compensation could be offered for the interview, or the time of treatment. Furthermore, the participants signed written informed consents, both in French and Kirundi, at the follow-up assessments consenting to their continued participation in the study and the anonymous use of the collected data for scientific purposes. They also consented in a separate written informed consent that parts of their anonymized narrations could be used for scientific purposes and trainings. The consents were read out and explained to participants who could not read. In the case of the underage participant, a guardian was asked to provide additional consent. No participant refused participation. For participating in the 9-months follow-up assessment the participants received a monetary compensation of 5000 BIF (~ 3 € in April 2015) to offset any monetary costs (travel, loss of wages) associated with their participation in the study. The Ethical Review Board of the *Université de Lumière of Bujumbura* approved this study. The study was registered at Clinical Trials: UKCR2014.

### Setting, procedure, and research design

This study was conducted between April 2014 and May 2015. The baseline assessment (baseline) started approximately 2 months after the flood disaster and was conducted in the emergency camps. The 3-months and 9-months follow-up assessments after the completion of NET were carried out in the participants’ new homes, where they had resettled approximately 6 months following the disaster. The interviewers ensured privacy during the assessments by selecting locations where the conversation could not be overheard and that were as quiet as possible. Such locations included tents provided for that purpose in the emergency camps, and church facilities during the baseline assessments, and the homes of the participants during later follow-ups. The vast majority of the sample suffered from considerable mental health symptoms. Due to the humanitarian purpose of the project, ethical considerations and limited resources, we chose the 15 individuals most affected by PTSD symptoms to receive NET (*NET group*). The remaining participants did not receive an intervention (*No Treatment group)* but were assessed at the same time points as the NET group in order to monitor the development of their symptoms in the aftermath of the disaster. Individuals in the NET group received 6 sessions, once per week, with each session lasting between 1.5 and 2.5 h depending on the needs of the participant. One participant was excluded because she discontinued the NET after the second session due to personal reasons and continued it only after the 3-months follow-up. Another participant who had received NET could not be relocated for the 3-months follow-up. We lost 9 individuals of the *No Treatment group* at the 3-months follow-up because their place of residence could not be located. Due to political riots in Bujumbura in spring 2015 the 9-months follow-up assessment could not be completed. However, prior to stopping the project for security reasons, contact with eight participants of the *NET group* and 10 participants of the *No Treatment* group had resumed.

Twelve psychology students from the University Lumière of Bujumbura carried out the interviews under the supervision of the authors of this article. All of the students were in their final academic year of their undergraduate studies. The interviewers had been extensively trained in the relevant concepts of mental disorders, in using the employed psychometric instruments and had acquired supervised practical experience in previous research projects. Six of the students conducted the baseline assessment and assisted in organizing the follow-up assessments by maintaining contact with the participants. We aimed to keep the interviewers of the follow-up assessments blind to whether the participants received NET or did not receive any treatment. Hence six additional students joined the team, three at the 3-months and three at the 9-months follow-up assessment. To ensure high quality interviews and rigorous oversight, each interview was discussed afterwards with one of the authors.

### NET training and supervision

The six students from the baseline-interview were selected to receive a six-day NET training by the first author in advance. The training emphasized the basic principals of NET and challenges in providing psychological assistance to people suffering from PTSD. Furthermore, the students were given time for practical exercises in groups to simulate sessions. After the successful completion of the training-course, each therapist received three clients. Five of the students carried out the NET interventions, the sixth student coordinated the activities and intervisions. Once a week, the therapies were supervised by the first author via skype. The trained students will be referred to as therapists in the remaining text.

### Instruments

The following instruments have been used for the assessments at baseline, 3- and 9-months follow-ups. All instruments have been translated and blindly back translated by the research team and local translators from the English/French versions [48] into Kirundi. Difficulties or uncertainties appearing in the process were discussed in detail amongst German and Burundian mental health experts prior to data collection to ensure proper adaptation to the Burundian context. The majority of the instruments have been previously used in clinical research projects in Burundi [49, 50]. WTP, stigmatization, and SES were only assessed at the follow-up assessments.

#### Socio-economic status (SES)

Participants were interviewed about their occupation prior to the flood and in the aftermath. Further, we assessed whether their earnings in the months prior to the follow-up assessments originated from donations, temporary work, or a more secure place of employment. The total amount of money available to the participants during each of these months was also recorded. In addition, the current living situation of each participant was assessed. They were asked whether they rented an apartment, owned property, lived on the streets, in a camp, or as a guest in another house and how much they paid or would have to pay for their accommodation. In addition, we asked how much money they had received from the Burundian Red Cross*.* We then calculated a mean value of the 3 months of income prior to the 3-months follow-up assessment.

#### Traumatic events

The number of traumatic event types experienced was assessed using a slightly adapted checklist that had already been used in a variety of contexts with populations affected by civil war, including Burundi [3, 50, 51]. The list consisted of 29 items assessing the lifetime trauma events load. Seven items assessed specifically traumatic events and maltreatment (physical violence, sexual violence, emotional violence, neglect) during childhood. Events from the Posttraumatic Diagnostic Scale [52] were incorporated as well as different war-related witnessed and self-experienced events. Items were coded dichotomously with 0 (no) or 1 (yes) and summed up.

#### PTSD symptom severity

The PTSD Symptom Scale – Interview (PSS-I*;* [53]) is a semi-structured interview and was used to determine *PTSD symptom severity*. The instrument is validated to assess symptoms of PTSD experienced in the previous month and due to the most traumatic event that occurred at least 1 month before the assessment. The response is rated on a 4-point scale by the interviewer. Four items were added to adhere to the DSM-V classification of PTSD. The PSS-I has been tested in various cultural settings [54, 55] and showed good psychometric properties, e.g.*,* inter-rater reliability = 0.93 [53, 56], and Cronbach’s α = .90; [55], including assessments conducted in Burundi (Cronbach’s α = .94; [50]). The PSS-I has a maximum possible score of 60.

#### Depression symptom severity

The Patient Health Questionnaire (PHQ-9; [57]) is a short, self-administered instrument which measures depression in adults. The PHQ consists of nine items which ask for the appearance of core symptoms of major depression disorder during the 2 weeks prior to assessment. The interviewer rates the severity of symptoms on a 4-point scale. The PHQ-9 has been previously tested in the Burundian context and showed good validity [58]. In the present study, PHQ-9 showed high internal consistency, as determined by Cronbach’s α of .84.

#### Suicidal tendency

We used Module C of version 6 of the Mini International Neuropsychiatric Interview (M.I.N.I.; [59]) to assess if participants reported a moderate or high risk of committing suicide. The M.I.N.I. is a short structured diagnostic interview for psychiatric disorders. It has been frequently used in a variety of cultures and settings [60].

#### Willingness to Pay (WTP)

Interviewers explained the concept of *WTP* to each participant. Further, the interviewers had been sensitized during their training to ensure that each participant understood that the responses they provided would not result in any negative consequence and that the amount of money attributed was only fictitious. The participants were then asked, “Knowing what you know of the beneficial effects of the therapeutic intervention regarding mental health symptoms today, what amount of money would you have been willing to reject in order to receive an intervention immediately.” Hence, we used the concept WTP to assess how much money the participants would have rejected retrospectively. Taking the low education level of the participants into account the interviewers were trained to provide examples of what the participants might have afforded for the amount of money they reported being willing to refuse in order to ensure the participants had a meaningful understanding of the value they assigned to receiving the treatment. The amount of money was measured in Burundian franc (BIF) and later converted into € with an exchange rate of 1661 BIF ~ 1 €.

#### Stigmatization due to symptoms/treatment

The Perception of Stigmatization by Others for Seeking Help (PSOSH; [61]) is a 5-item instrument to measure social and public stigma, as a result of seeking psychological help. The participants are asked how they expected people to react regarding their mental health symptoms, or when they would seek psychological treatment. Five possible reactions were offered including the following questions: “a)… react negatively towards you; b)… have a bad opinion about you; c)… perceive you as seriously imbalanced; d)… think of you in a less favourable way; e)… think that you represent a risk to another person?” Their responses were rated on a 5-point Likert-scale ranging from not at all (1) to a great deal (5). A higher perception of stigmatization is then translated into a higher sum score (5–25). The PSOSH has been tested in patients with PTSD and showed good validity and reliability (e.g. Cronbach’s alpha = .91, test-retest reliability = .82; [62, 63]). Of note is that the PSOSH has been developed for the field of school counselling. We adapted the original phrase for the purpose of this study to either assess stigmatization experienced or expected due to symptoms of PTSD or due to seeking treatment.

### Data analysis

Data management and analysis were conducted using SPSS 20.0 [64] and R-Statistics [65]. There were no outliers and the data was normally distributed, as assessed by the Shapiro-Wilk test (*p* > .05). All further assumptions were met, if not mentioned otherwise below. Repeated measure analysis (ANOVAs) were performed with time as a 3-level within-subject variable (baseline, 3 and 9-months follow-up) and treatment groups as 2-level between-subjects variable (*NET group, No Treatment group*). Due to the high dropout rate resulting from the political instability in spring 2015, we also conducted a repeated ANOVA including only the 3-months follow-up assessment to confirm the results. Because of the directional hypotheses regarding the effects of the intervention, and improvement of symptoms over time in the *No Treatment group*, analyses of interaction and time effects were computed one-tailed on an alpha level of .05. The effect size for pairwise comparisons was estimated using Hedges’ *g* (Hedges, 1981). *Hedges’ g* was considered small with *Hedges’ g* ≥ 0.20, moderate with *Hedges’ g* ≥ 0.50, and large with *Hedges’ g* ≥ 0.80. To provide statistics for changes on the individual level, we calculated *reliable change indices* (*RCIs*; [66]) for changes in *PTSD symptom severity* and *depression symptom severity*. As a result of directional hypotheses, *RCI*-values greater than 1.65 indicated a significant difference.

As neither *WTP* nor *SES* were normally distributed (Shapiro-Wilk test: *p* < .05) and homogeneity of variances (Levene test: *p* < .001) was lacking amongst the treatment groups, we used Mann-Whitney U and Wilcoxon-sign rank tests for comparisons. We tested whether the treatment groups differed in *WTP* and income at the 3-months follow-up. Furthermore, in the *NET group* we assessed if *WTP* changed between the follow-ups. Comparing *stigmatization due to symptoms* with *stigmatization due to intervention* at the 3-months follow-up, we conducted paired t-tests for each of the treatment groups. Furthermore, we assessed if the *stigmatization due to symptoms* reduced between the follow-ups in the *NET group*. Controlling for multiple comparisons we used the Bonferoni-Holm procedure. We then calculated a spearman-correlation to assess potential relationships between *PTSD symptom severity, stigmatization due to symptoms, and stigmatization due to intervention*.

## Results

### Descriptive statistics at baseline

The mean age of our sample was 28 years (*SD =* 11.7 [14 to 78]). Approximately one third of the participants were illiterate (*n* = 11, 37.9%). In total participants had received an average of 4 years of education (*M* = 3.8, *SD* = 2.5). The average income across all participants for the first 3 months after successful completion of the therapy was 33.5 € (*SD* = 26.4) per month. There was no significant difference between treatment groups in any of the socio-demographic or socio-economic characteristics at baseline assessment, as assessed by χ2 and t-tests (Tables 1 and 2).

**Table 1:** Socio-demographic data and outcome assessments at pretest

		No Treatment				Narrative Exposure Therapy				Total							
		*M*	*SD*	*n*	*%*	*M*	*SD*	*n*	*%*	*M*	*SD*	*n*	*%*	*X* ^*2*^	*p*	*T*	*p*
Age in years		29.8	14.9			26.3	5.8			28.2	11.7					0.87	.40
Sex	Male			2	12.5			3	23.0			5	17.2	0.07	.80		
	Female			14	87.5			10	77.0			24	82.7				
Illiteracy	No			10	62.5			8	61.5			18	62.1	0.00	.99		
	Yes			6	37.5			5	38.5			11	37.9				
Education in years		3.9	2.1			3.7	2.9			3.8	2.5					0.25	.80
Marital status	Single			3	18.8			3	23.0			6	20.7	4.65	.33		
	Married			6	37.5			2	15.4			8	27.6				
	Divorced			1	6.3			0	0.0			1	3.5				
	Widowed			1	6.3			4	30.8			5	17.2				
	In relationship			5	31.3			4	30.8			9	31.0				
Number of children lost	0			8	50.0			3	23.1			11	37.9	3.78	.44		
	1			4	25.0			5	38.5			9	31.0				
	2			2	12.5			4	30.8			6	20.7				
	3			1	6.3			1	7.6			2	6.9				
	4			1	6.3			0	0.0			1	3.5				
Occupation before flood disaster	None			2	12.5			0	0.0			2	6.9	3.12	.54		
	Student			1	6.3			0	0.0			1	3.5				
	Some job			13	81.3			13	100.0			27	89.6				
Occupation after flood disaster	None			1	6.3			2	15.4			3	10.3	2.25	.69		
	Student			1	6.3			0	0.0			1	3.5				
	Some job			14	87.5			11	84.6			25	86.2				
Traumatic events		16.9	5.4	16		14.0	4.4	13		18.0	4.9	29				1.43	.17
Depression symptom severity^***^		9.1	3.2	16		16.0	4.2	13		12.2	5.1	29				4.88	<.001
PTSD symptom severity ^***^		20.3	5.7	16		35.9	5.7	13		27.3	9.7	29				7.29	<.001

***indicate significant differences with *p* ≤ .001

**Table 2:** Socio-economic data

		No Treatment				Narrative Exposure Therapy				Total						
		*M*	*SD*	*n*	*%*	*M*	*SD*	*n*	*%*	*M*	*SD*	*n*	*%*	*X* ^*2*^	*T*	*p*
Average income per month in the first 3 months after treatment completion (€)		31.5	22.9			36.0	31.0			33.5	26.4				0.44	.67
Costs housing 3-months follow-up (€)		12.6	6.2			14.8	5.8			13.3	6.0				0.72	.49
Housing condition 3-months follow-up	Rent			12	75.0			7	53.8			19	65.5	0.64		.42
	Property			0	0.0			0	0.0			0	0.0			
	Guest			4	25.0			6	46.2			10	34.5			
	On the street			0	0.0			0	0.0			0	0.0			
	In a camp			0	0.0			0	0.0			0	0.0			

As illustrated in Table 1 participants within the *NET group* suffered severely from PTSD symptoms (*M* = 35.9, *SD* = 5.7) and from depression symptoms (*M* = 16.0, *SD* = 4.2). Five participants reported moderate to severe suicidal tendencies at pretest. The *No Treatment group* was less affected than the *NET group* but still reported significant *PTSD symptom severity* (*M* = 20.3, *SD* = 5.7) and *depression symptom severity* (*M* = 9.1, *SD* = 3.2). Three *No Treatment group* participants reported moderate to severe suicidal tendencies. Participants of both groups reported a similar amount of traumatic events experienced throughout lifetime.

### Mental health symptoms

As the assumption of sphericity for the *PTSD symptom severity* was violated, we corrected the degrees of freedom using Greenhouse-Geisser estimates of sphericity. For the repeated measures ANOVA, we found a statistically significant time (baseline, 3-months, 9-months) x group (*NET group, No Treatment group*) interaction (*F*(1.40, 22.38) = 4.29, *p* = .011, η^2^_p_ = .14). Furthermore, we found a significant main effect of time, *F*(1.40, 22.38) = 38.47, *p* < .001, η^2^_p_ = .60), and a non-significant main effect of group, *F*(0.70, 11.19) = 2.06, *p* = .17, η^2^_p_ = .05). Overall, the *PTSD symptoms severity* improved significantly in both groups from baseline to 3-months follow-up (*Hedges’ g*_*NET*_ *=* 1.62; *Hedges’ g*_*No Treatment*_ *=* 0.64) and from baseline to 9-months follow-up (*Hedges’ g*_*NET*_ *=* 3.44; *Hedges’ g*_*No Treatment*_ *=* 2.55). Overall, the improvements were more pronounced in the *NET group*. On the individual level the RCIs indicated that a high percentage of participants in the *NET group* suffered significantly less from PTSD symptoms (75%) or reported an unchanged symptom severity (25%) at 3-months follow-up, while 100% of them had significantly improved at 9-months follow-up. The majority of the participants of the *No Treatment group* suffered less (60%), while 30% remained unchanged, and 10% deteriorated regarding symptom severity at 3-months follow-up. At 9-months follow-up, 80% of the *No Treatment group* had improved significantly, and 20% had remained unchanged.

**Fig. 2:**
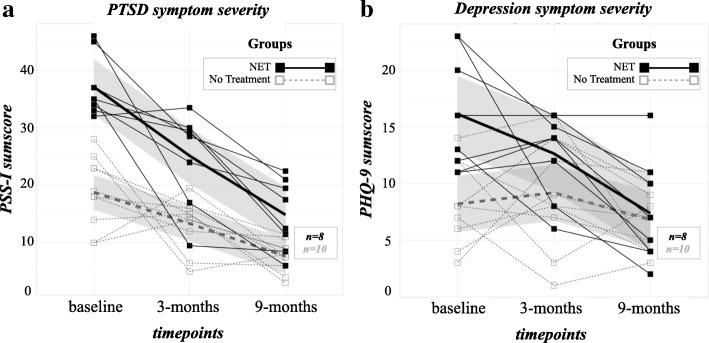
Spaghetti plots of (**a**) PTSD symptom severity and (**b**) Depression symptom severity at baseline, 3-months and 9-months follow-up for the individuals in the Narrative Exposure Therapy group (in black) or the No Treatment group (in dashed grey) respectively. *Thicker lines r*epresent the linear model, *Grey shaded areas* represent the standard error

Further, we calculated a second repeated measures ANOVA for the 3-months follow-up to account for the participant attrition due to the political instability in Burundi. We found a significant interaction of time (baseline, 3-months) x group (*NET group, No Treatment group*; *F*(1, 27) = 4.07, *p* = .027, *η*^*2*^_*p*_ = .07), a significant main effect of time (*F*(1, 27) = 20.68, *p* < .001; *η*^*2*^_*p*_ = .27), and a significant main effect of group (*F*(1, 27) = 38.25, *p* < .001; *η*^*2*^_*p*_ = .43) (Fig. 2).

In the repeated measures ANOVA regarding *depression symptom severity*, we found a statistically significant time (baseline, 3-months, 9-months) x group (*NET group, No Treatment group*) interaction (*F*(2, 32) = 4.59, *p* = .009, *η*^*2*^_*p*_ = .13). Furthermore, we found a significant main effect of time, (*F*(2, 32) = 9.35, *p* < .001, *η*^*2*^_*p*_ = .23), and a significant main effect of group (*F*(1, 16) = 8.26, *p* = .011, *η*^*2*^_*p*_ = .20). Overall, the *depression symptom severity* improved significantly in the *NET group* from baseline to 3-months follow-up (*Hedges’ g*_*NET*_ *=* 0.84; *Hedges’ g*_*No Treatment*_ *=* − 0.06) and from baseline to 9-months follow-up in both groups (*Hedges’ g*_*NET*_ *=* 1.88; *Hedges’ g*_*No Treatment*_ *=* 0.72). Overall, the improvements were more pronounced in the *NET group*. On the individual level the RCIs indicated that a high percentage of participants of the *NET group* suffered significantly less from depression symptoms (50%), while some reported an unchanged symptom severity (37.5%), and a minority reported deteriorated symptom severity (12.5%) at 3-montsh follow-up, while 100% of them had improved significantly at 9-months follow-up. Approximately one-third of the participants of the *No Treatment group* suffered less (30%) from depression symptoms, while another third remained unchanged (30%), and the majority (40%) deteriorated regarding symptom severity at 3-months follow-up. At 9-months follow-up, the *depression symptom severity of* 50% of the *No Treatment group* had improved significantly, while the symptoms of 30% had remained unchanged, and of 20% had deteriorated significantly.

Further, we calculated a second repeated measures ANOVA for the 3-months follow-up to encounter the drop-out due to the political instability in Burundi. We found a significant interaction of time (baseline, 3-months) x group (*NET group, No Treatment group*; *F*(1, 27) = 3.57, *p* = .035, *η*^*2*^_*p*_ = .06), a non-significant main effect for time (*F*(1, 27) = 2.71, *p* < .055, *η*^*2*^_*p*_ = .04), and a significant main effect for group, (*F*(1, 27) = 20.28, *p* < .001, *η*^*2*^_*p*_ = .429).

### WTP and monthly income

Assessing *WTP* and monthly income, we excluded one participant in the *NET group* at 3-months follow-up because of missing data. In total we included 28 participants (*n*_*NET*_ = 12; *n*_*No Treatment*_ = 16) for the 3-months and 18 participants (*n*_*NET*_ = 7; *n*_*No Treatment*_ = 10) for the 9-months follow-up.

**Fig. 3:**
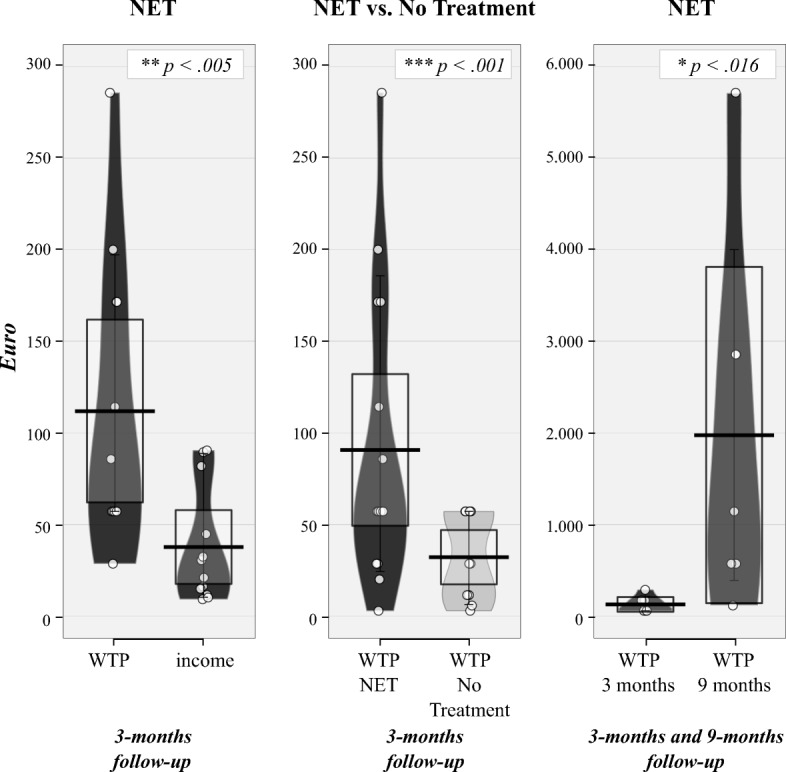
RDI (*Raw* data, *Descriptive* and *Inferential* statistics) plots of average income and Willingness To Pay (WTP) at 3-months and 9-months follow-up for the individuals in the Narrative Exposure Therapy group or the No Treatment group respectively. *Dots* represent the raw data, vertical *black bar* shows central tendency, *bean* representing a smoothed density, *whisker* representing 95 confidence interval

As illustrated in Fig. 3, *WTP* was significantly higher than the monthly income for participants in the *NET group* at 3-months follow-up (*W* = 73.0, *z* = − 2.85, *p* < .005). In addition, *WTP* in the *NET group* was significantly higher at 9-months compared to 3-months follow-up (*W* = 28.0, *z* = − 2.418, *p* = .016). Compared with the *No Treatment group*, the *NET group* reported a significantly higher *WTP* at 3-months follow-up (*U* = 22.5, *z* = − 3.504, *p* < .001). Still, the *No Treatment group* reported a *WTP* comparable to their monthly income to receive the intervention immediately (*W* = 68.0, *z* = 0.0, *p* = 1.0).

### Stigmatization due to symptoms of PTSD & seeking treatment

**Fig. 4:**
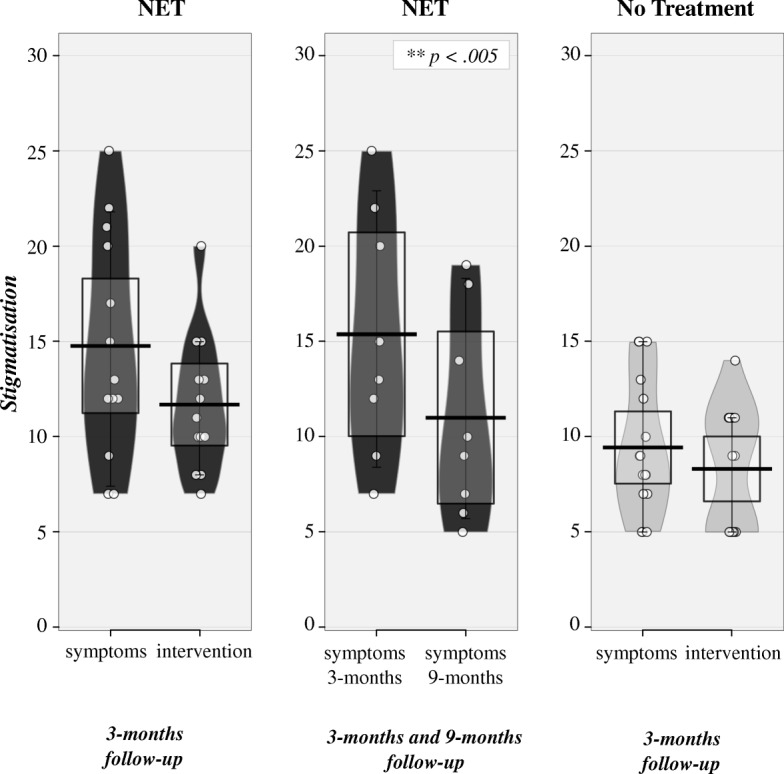
RDI (*Raw* data, *Descriptive* and *Inferential* statistics) plots of average stigmatization concerning symptoms and intervention at 3-months and 9-months follow-up for the individuals in the Narrative Exposure Therapy group or the No Treatment group respectively. *Dots* represent the raw data, vertical *black bar* shows central tendency, *bean* representing a smoothed density, *whisker* representing 95 confidence interval

As illustrated in Fig. 4, we found a non-significant mean difference of *stigmatization due to symptoms* and *stigmatization due to treatment* in the NET group at 3-months follow-up (*t*(12) = 2.08, *p* = .0595, Hedges*’ g* = 0.62). However, a trend toward significance could be observed with a medium effect size: Participants of the NET group reported more *stigmatization due to symptoms* (*M* = 14.77, *SD* = 5.83) than *stigmatization due to treatment* (*M* = 11.69, *SD* = 3.57). The *stigmatization due to symptoms* reduced between the 3-months (*M* = 15.38, *SD* = 6.39) and the 9-months follow-up (*M* = 11.00, *SD* = 5.40) within the NET group (*t*(7) = 4.162, *p* = .004, *Hedges’ g* = 0.70). The *No Treatment group* did not differ regarding *stigmatization due to symptoms* (*M* = 9.44, *SD* = 3.56) and expected *stigmatization due to treatment* (*M* = 8.33, *SD* = 3.20) at 3-months follow-up (*t*(15) = 0.98, *p* = .34, *Hedges’ g* = 0.32). Within the *NET group*, *PTSD symptoms severity* correlated significantly with *stigmatization due to symptoms* at 3-months (*r*(11) = .55, *p* = .051) and 9-months (*r*(6) = .71, *p* = .048) follow-up and did not correlate significantly with *stigmatization due to intervention* at 3-months (*r*(11) = 0.21, *p* = .49) or 9-months follow-up (*r*(6) = 0.57, *p* = .14).

## Discussion

The present paper provided evidence for the feasibility of conducting an evidence based, trauma-focused exposure therapy such as NET with survivors of natural disasters in a post-conflict region while they are still early in the recovery process. Symptom severity of PTSD (*Hedges’ g*_*NET*_ *=* 3.44; *Hedges’ g*_*No Treatment*_ = 2.55) and depression (*Hedges’ g*_*NET*_ *=* 1.88; *Hedges’ g*_*No Treatment*_ = 0.72) improved significantly in both groups at 9-months follow-up with a greater improvement amongst those participants who had received NET. In addition, participants in the NET group assigned great value to the psychological intervention by indicating their willingness to refuse a sum equal to 1 month’s income (35 €) at 3-months and a sum equal to more than a year of income at 9-months follow-up to receive such an intervention immediately in the aftermath of the disaster. Finally, we found a significant decrease of stigmatization due to symptoms of PTSD in the NET group between the 3-months and the 9-months follow-up, indicating that addressing mental health issues using evidence-based treatment most likely improves the well-being of individuals by reducing or even removing daily stressors such as stigmatization.

In the aftermath of the flood disaster, we relied on volunteers of the Burundian Red Cross to identify survivors they considered most mentally affected according to the following three criteria: (1) having lost a close relative; (2) being isolated or behaving strangely in the camp; and (3) hearing about symptoms such as nightmares, sleeping troubles, or angry outbursts. Following our screening criteria, 40 out of 51 survivors we enrolled fulfilled enough symptoms to diagnose them with PTSD. This result suggests that identifying affected survivors is feasible in emergency camps and can be carried out by briefed volunteers. Furthermore, it might reflect a generally high prevalence of trauma-related mental health disorders in such regions.

Survivors in the NET group suffered from severe symptoms of PTSD and depression and often experienced multiple traumatic experiences besides the natural disaster, including events related to the civil war. We were able to replicate the beneficial effect of NET regarding mental health symptoms in the aftermath of a natural disaster [23, 24]. As in many other studies (for an overview see [25]), we found the strongest improvement of mental health symptoms after a longer period of time had passed. This finding is usually attributed to the idea that during NET a memory process is started which continues over the following months and years and causes an ongoing improvement of symptoms. This process is related to dissolving the fear network through neural changes due to elaboration and overcoming avoidance behaviour. The sustainable effect of NET has been well-established over the past 15 years and is considered a particular strength of this approach [30, 32].

The beneficial effect of NET within this sample was found even though both follow-up assessments were conducted at the respective beginning of the two rainy seasons in Burundi. Particularly, the rain falling during 3-months follow-up assessment triggered many memories and elicited past fears among the participants. After the closure of the emergency camps, the majority of participants moved back to the same suburb of Bujumbura where they had resided prior to the flood disaster. Many survivors were therefore exposed to locations and cues related to the loss of their loved ones. Furthermore, many of the participants struggled with their grief associated with the death of their children. Shortly prior to our first follow-up assessment, the Burundian government had conducted a memorial service for all families who had lost their children during the flood disaster. Unfortunately, two of the participants within the NET group reported that they had not been officially recognized as parents who had lost their children, which caused increased mental health symptoms and even suicidal ideations. Due to ethical considerations we offered some support monitoring their suicidal ideations and conducted a memorial with those two participants to acknowledge their losses thereby assisting them to overcome those ideations.

The PTSD and depression symptoms of participants less affected from trauma-related mental health disorders in the aftermath of the natural disaster also improved significantly over time. This result indicates that less severely affected individuals might not require a trauma-specific intervention but might benefit from spontaneous remission when they slowly regain the previous standard of living. Overall, the mental health improvements of both groups, the no treatment and the intervention group, are in line with the assumptions that more severely affected individuals with an elevated risk of chronicity of PTSD symptoms [67], might benefit from trauma-focused interventions while less affected individuals might recover spontaneously. However, due to limitations of the study’s design we can not rule out the possibility that the less affected individuals might also have profited from NET, nor that the more severely affected individuals might have recovered spontaneously.

As in previous studies [26, 28], we demonstrated that the dissemination of NET to relatively inexperienced local counsellors is feasible within a short training period and yields very promising results regarding mental health benefits. In addition, we argue that the use of local counsellors counteracts stigmatization and supports the aim of NET of being aware of local traditions and cultural characteristics. These factors along with the fact that local counsellors advocated the intervention might have improved the acceptance amongst the beneficiaries.

At 3-months follow-up, participants in both groups were willing to retrospectively refuse a sum equal to at least the income of 1 month in order to receive an individual trauma-focused intervention while still living in the emergency camps. In the NET group, the amount was significantly higher and increased over time.This result emphasizes the argument made by Schauer and Schauer [15] that providing “appropriate mental health services for trauma victims … [is] anything but a “luxury” especially in resource poor, conflict ridden countries ([16], 4-5, pp.).” Furthermore, the quantitative results are in line with various qualitative accounts of traumatized survivors across different cultures whose testimonies provide evidence that narrating and acknowledging their stories through the process of NET is highly appreciated ([16, 68, 69], 4-5, pp.). We can only speculate if the participants’ high WTP is mainly related to improved mental health [25] including overcoming survivor's guilt, shame, and feelings of revenge [11, 70], or improved status due to improved functioning [6, 71], or both. Nevertheless, the amounts reported indicate that this population is aware of the impairment they suffer due to mental health problems and that they are assigning a significant value to receiving treatment. The fact that the relative WTP of our sample (100% of monthly income) was much higher than the WTP of a sample of depressed American patients (9% of monthly income) [37] might be attributed to differences of the samples regarding general income and living conditions, culture, diagnostics, and to the fact that we asked how much money our participants would have refused in order to receive treatment as soon as possible instead of asking them to pay that amount. However, it might also emphasize how much appropriate mental health treatments are valued in post-conflict countries.

Stigmatization and resentment due to competition regarding scarce resources present a serious obstacle to psychological interventions in emergency camps. Even more so, as there is almost no privacy in such circumstances and others notice when someone receives extra attention in the form of a therapeutic intervention in the camps. In light of this, we provided psychoeducation to the community in order to counter this obstacle to participation in the treatment. Over the course of the intervention, all but one participant completed the NET while living in the emergency camps. Furthermore, participants of both groups indicated at the first follow-up that they did not or did not expect to feel more stigmatized due to receiving trauma-focused treatment than they felt stigmatized due to their symptoms. The NET group even reported that they felt less stigmatized by the fact that they had received treatment than because of their symptoms. The continuous association between PTSD symptoms and feeling stigmatized indicates that stigmatization due to symptoms most likely severely affects the social status of individuals in their community and hence elicit ongoing suffering. Overall, these results strongly contradict the idea that proactively addressing mental health problems in severely affected populations living in circumstances that offer little privacy would aggravate the situation of affected individuals due to stigmatization. Instead, addressing mental health issues using evidence-based treatment most likely improves the well-being of individuals by removing daily stressors in the form of stigmatization [14].

The presented study has the following caveats. The non-randomized design, the small sample size, the loss of participants during the follow-ups, and the possible regression to the mean are confounding factors. It is possible that the within group pre-post differences and the between group differences of the mental health symptoms arise from those factors. Hence the conclusions regarding the development of mental health symptoms have to be considered with a grain of salt. According to a post-hoc sample size calculation, assuming Bonferoni corrected significance for testing PTSD and depression levels, and a power of .8, future studies aiming to replicate the mental health related effects of NET in the aftermath of a natural disaster would require a total sample size of at least 16 participants. Regarding the feasibility of dissemination of the NET approach to local counsellors in short trainings, we must acknowledge that a 6-day training is unusually short. Such a short training was only feasible because the therapists already had extensive experience in mental health diagnostics, and had some background knowledge and experience due to their study of psychology in Burundi. Moreover, we would like to stress the necessity of supervision for newly trained therapists as they are quickly confronted with very severe cases including dissociation and suicidal ideations.

It is important that we address our attempt to operationalize cultural acceptance via the concept of WTP. We are aware that the concept of assigning a monetary value to assess the subjectively-perceived benefits of an intervention itself might be a new and maybe even capitalistic idea. Furthermore, this approach is arguably reducing the benefits of a therapy to a single dimension. As the amounts of money were fictional we cannot rule out that the participants exaggerated the amount they would have been willing to refuse in retrospect.

## Conclusions

Implementing such a project in emergency camps about 2 months after the disaster required us to consider the following obstacles: (1) Potential stigmatization and resentment because of the extra attention some of the survivors received due to our intervention; (2) rumours and worries including ideas about witchcraft we might use when talking in private with the participants; and (3) frustration and confusion about psychological interventions in general. According to accounts of Red Cross volunteers and of survivors in the emergency camps, the psychological response of institutions and NGOs had not been well-coordinated within the emergency camps. The different approaches, lack of transparency and psychoeducation had confused the survivors in the camps and resulted in mistrust towards psychologists.

While navigating these challenges, we demonstrated that implementing specialized trauma-focused treatment in the aftermath of disaster is feasible in post-conflict regions. NET has been confirmed as a promising approach for severely affected individuals given that it can be disseminated to lay counsellors, both directly and via train-the-trainer models [28]. At the same time our experiences during the implementation of the project indicate the need for (1) reinforcing and expanding coordinated mental health approaches for interventions in the aftermath of disasters in order to to combine activities of NGOs instead of diffusing impact due to confusion created by different approaches without coordination; (2) evaluating interventions in this context; (3) implementing evidence-based trauma-focused interventions while also addressing grief and ideas of revenge that arose in some survivors as they tried to deflect blame and attribute meaning to their losses; and (4) building up mental health capacities in post-conflict countries such as Burundi. The results indicated furthermore that NET is well received by the affected populations and that NET might contribute significantly to reducing daily stressors associated with stigmatization due to PTSD.

## Abbreviations

NET: Narrative Exposure Therapy
PTSD: Posttraumatic stress disorder
SES: Socio economic status
WTP: Willingness to pay
